# Diurnal Fluctuations in Steroid Hormones Tied to Variation in Intrinsic Functional Connectivity in a Densely Sampled Male

**DOI:** 10.1523/JNEUROSCI.1856-23.2024

**Published:** 2024-04-16

**Authors:** Hannah Grotzinger, Laura Pritschet, Pavel Shapturenka, Tyler Santander, Elle M. Murata, Emily G. Jacobs

**Affiliations:** ^1^Departments of Psychological & Brain Sciences, University of California, Santa Barbara, California 93106; ^2^Chemical Engineering, University of California, Santa Barbara, California 93106; ^3^Neuroscience Research Institute, University of California, Santa Barbara, California 93106

**Keywords:** brain structure, diurnal rhythms, MRI, precision imaging, steroid hormones

## Abstract

Most of mammalian physiology is under the control of biological rhythms, including the endocrine system with time-varying hormone secretion. Precision neuroimaging studies provide unique insights into how the endocrine system dynamically regulates aspects of the human brain. Recently, we established estrogen's ability to drive widespread patterns of connectivity and enhance the global efficiency of large-scale brain networks in a woman sampled every 24 h across 30 consecutive days, capturing a complete menstrual cycle. Steroid hormone production also follows a pronounced sinusoidal pattern, with a peak in testosterone between 6 and 7 A.M. and nadir between 7 and 8 P.M. To capture the brain's response to diurnal changes in hormone production, we carried out a companion precision imaging study of a healthy adult man who completed MRI and venipuncture every 12–24 h across 30 consecutive days. Results confirmed robust diurnal fluctuations in testosterone, 17β-estradiol—the primary form of estrogen—and cortisol. Standardized regression analyses revealed widespread associations between testosterone, estradiol, and cortisol concentrations and whole-brain patterns of coherence. In particular, functional connectivity in the Dorsal Attention Network was coupled with diurnally fluctuating hormones. Further, comparing dense-sampling datasets between a man and a naturally cycling woman revealed that fluctuations in sex hormones are tied to patterns of whole-brain coherence in both sexes and to a heightened degree in the male. Together, these findings enhance our understanding of steroid hormones as rapid neuromodulators and provide evidence that diurnal changes in steroid hormones are associated with patterns of whole-brain functional connectivity.

## Significance Statement

Diurnal variation is an essential biorhythm, yet the relationship between diurnal fluctuations in steroid hormones and the functional architecture of the human brain is virtually unknown. This precision neuroimaging study suggests that endogenous fluctuations in testosterone, estradiol, and cortisol concentrations are tied to rhythmic changes in coherence across the brain. Precision imaging studies that track individuals across endocrine rhythms (e.g., the diurnal cycle and menstrual cycle) demonstrate steroid hormones’ ability to modulate the functional architecture of the brain in both sexes and provide a roadmap for future studies to probe the functional significance of these rhythms for behavior.

## Introduction

The mammalian brain is densely packed with steroid hormone receptors, yet the extent to which these signaling molecules influence the large-scale functional architecture of the human brain is remarkably understudied. At the cellular level, steroid hormones regulate synaptic plasticity in cortical and subcortical regions (Galea et al., 2017; Taxier et al., 2020). At the macro-scale, hormonal transitions such as puberty (McDermott et al., 2012; Pattwell et al., 2013; Brouwer et al., 2015), the menstrual cycle (Pritschet et al., 2020; Taylor et al., 2020; Zsido et al., 2023), pregnancy (Hoekzema et al., 2017; Carmona et al., 2019; Pritschet et al., 2023, bioRxiv; Paternina-Die et al., 2024), menopause (Jacobs et al., 2017), and andropause (Janowsky, 2006) are characterized by changes in brain structure and function. Hormonal fluctuations across shorter timescales, like diurnal rhythms, may also influence the brain. Previous neuroimaging studies have examined brain activity related to circadian rhythms (Gaggioni et al., 2014; Jiang et al., 2016; Oka et al., 2024), as well as the impact of disrupting circadian rhythms on brain function, including sleep deprivation (Fang and Rao, 2017), exposing individuals to blue light (Schoonderwoerd et al., 2022), and psychopathology (McKenna et al., 2014; Chen et al., 2022). However, relationships between diurnal variation in sex steroid hormones and human brain network organization are virtually unknown.

Human brain imaging studies often draw inferences about hormone–brain relationships via cross-sectional designs, where subjects are observed at one or two discrete hormonal states. However, a key feature of the mammalian endocrine system is that hormone secretion varies continuously over time. Cross-sectional snapshots of the brain at one timepoint obscure the true dynamic range of brain–hormone interactions unfolding across periodic rhythms. This challenge can be addressed by precision imaging studies, which densely sample individuals over time (Poldrack et al., 2015; Gordon et al., 2017; Fedorenko, 2021). Leveraging this approach to track the brain across the menstrual cycle has generated novel insight into the role our endocrine system plays in regulating dynamic, rhythmic changes in the brain (Fitzgerald et al., 2020; Pritschet et al., 2020, 2021; Taylor et al., 2020; de Filippe et al., 2021; Mueller et al., 2021; Zsido et al., 2023; Jacobs, 2023).

Previously, our group examined the relationship between sex hormones and functional brain networks in a woman sampled every 24 h for 30 consecutive days (Fitzgerald et al., 2020; Pritschet et al., 2020; Taylor et al., 2020; Mueller et al., 2021). Across the menstrual cycle, rhythmic changes in 17-β estradiol were found to drive increases in functional coherence and enhance global efficiency in several intrinsic brain networks, including the Default Mode, Dorsal Attention, and Temporal Parietal networks—notably, these networks consist of hubs populated with sex hormone receptors. These findings provided a foundation for understanding estradiol-driven changes in large-scale brain network organization but lacked a complementary dataset to test these associations in a densely sampled man.

Many people undergo rhythmic changes in hormone production over a ∼28 d reproductive cycle. Most people also experience a diurnal cycle: testosterone concentrations peak in the morning and decline by ∼50% or more throughout the day (Rose et al., 1972; Barberia et al., 1973; Diver et al., 2003), with estradiol and cortisol production following suit (∼35 and ∼85%, respectively; Berg and Wynne-Edwards, 2001; Ankarberg-Lindgren and Norjavaara, 2008). To determine the influence of these diurnal hormone fluctuations on functional brain networks, we used a dense-sampling design to collect (f)MRI, serum, saliva, and mood data from a healthy adult male every 12–24 h for 30 consecutive days. Briefly, day-to-day changes in testosterone, estradiol, and cortisol show widespread associations with cortical network dynamics, particularly dorsal and ventral attention networks. Comparing dense-sampling datasets by sex reveals that fluctuations in testosterone and estradiol are tied to patterns of whole-brain coherence to a greater degree in our male participant. Together, findings from this study enhance our understanding of steroid hormones as rapid neuromodulators and provide evidence that diurnal changes in hormone production impact the brain's functional network architecture.

## Materials and Methods

### Participant

The participant was a 26-year-old right-handed Caucasian male with no history of neuropsychiatric diagnosis, endocrine disorders, or prior head trauma. The participant gave written informed consent for a study approved by the University of California, Santa Barbara Human Subjects Committee and was paid for their participation in the study.

### Experimental design

**Figure 1.**
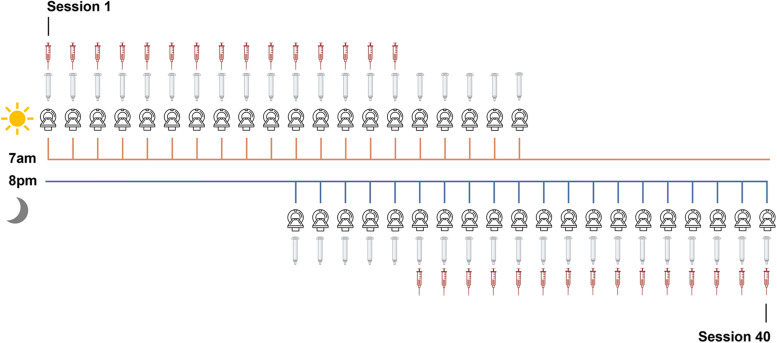
Sampling rate of MRI, venipuncture, and saliva acquisition. Forty time-locked sessions were performed over 30 consecutive days; *n* = 20 at 7 A.M. and *n* = 20 at 8 P.M. for a total of 40 sessions.

The methods for this study parallel those reported in Pritschet et al. (2020). The participant underwent venipuncture and brain imaging every 12–24 h for 30 consecutive days. At each session, the participant completed a daily questionnaire (see below, Behavioral assessments), followed by endocrine sampling at 7 A.M. (morning sessions) and at 8 P.M. (evening sessions). The participant gave a 2 ml saliva sample at each session, followed by a blood sample. On days with two test sessions (Days 11–20), the participant underwent one blood draw per day (Fig. 1) per safety guidelines (half A.M., half P.M.). Saliva provided a secondary assessment of steroid hormones. Morning endocrine samples were collected after at least 8 h of overnight fasting, and evening endocrine samples were collected following an hour and a half of abstaining from consumption of food or drink (excluding water). The participant refrained from consuming caffeinated beverages before each morning session.

#### Endocrine procedures

A ∼2 ml saliva sample was obtained via passive drooling into a wide mouthed plastic cryovial. The participant refrained from eating and drinking (besides water) at least 1.5 h before saliva sample collection, and morning samples were collected after fasting overnight. The participant pooled saliva for ∼5–10 min before depositing the sample into the cryovial to determine total testosterone and cortisol concentrations. The sample was stored at −20°C until assayed. Saliva concentrations were determined via enzyme immunoassay at Brigham and Women's Hospital Research Assay Core.

Immediately after the saliva sample was obtained, a licensed phlebotomist inserted a saline-lock intravenous line into either the dominant or nondominant hand or forearm daily to evaluate total testosterone, free testosterone, cortisol, and 17β-estradiol concentrations. Note that estradiol and free testosterone measurements were acquired only from serum samples, resulting in 30 timepoints. One 10cc ml blood sample was collected in a vacutainer SST (BD Diagnostic Systems) each session. The sample clotted at room temperature for ∼60 min until centrifugation (2,100 × *g* for 10 min) and was then aliquoted into three 2 ml microtubes. Serum samples were stored at −20°C until assayed. Serum concentrations were determined via liquid chromatography mass spectrometry at the Brigham and Women's Hospital Research Assay Core. Assay sensitivities, dynamic range, and intra-assay coefficients of variation (respectively) were as follows: estradiol, 1 pg/ml, 1–500 pg/ml, <5% relative standard deviation (RSD); testosterone, 1.0 ng/dl, 1–200 ng/dl, <2% RSD; cortisol, 0.5 ng/ml, 0.5–250 pg/ml, <8% RSD.

#### Behavioral assessments

**Figure 2.**
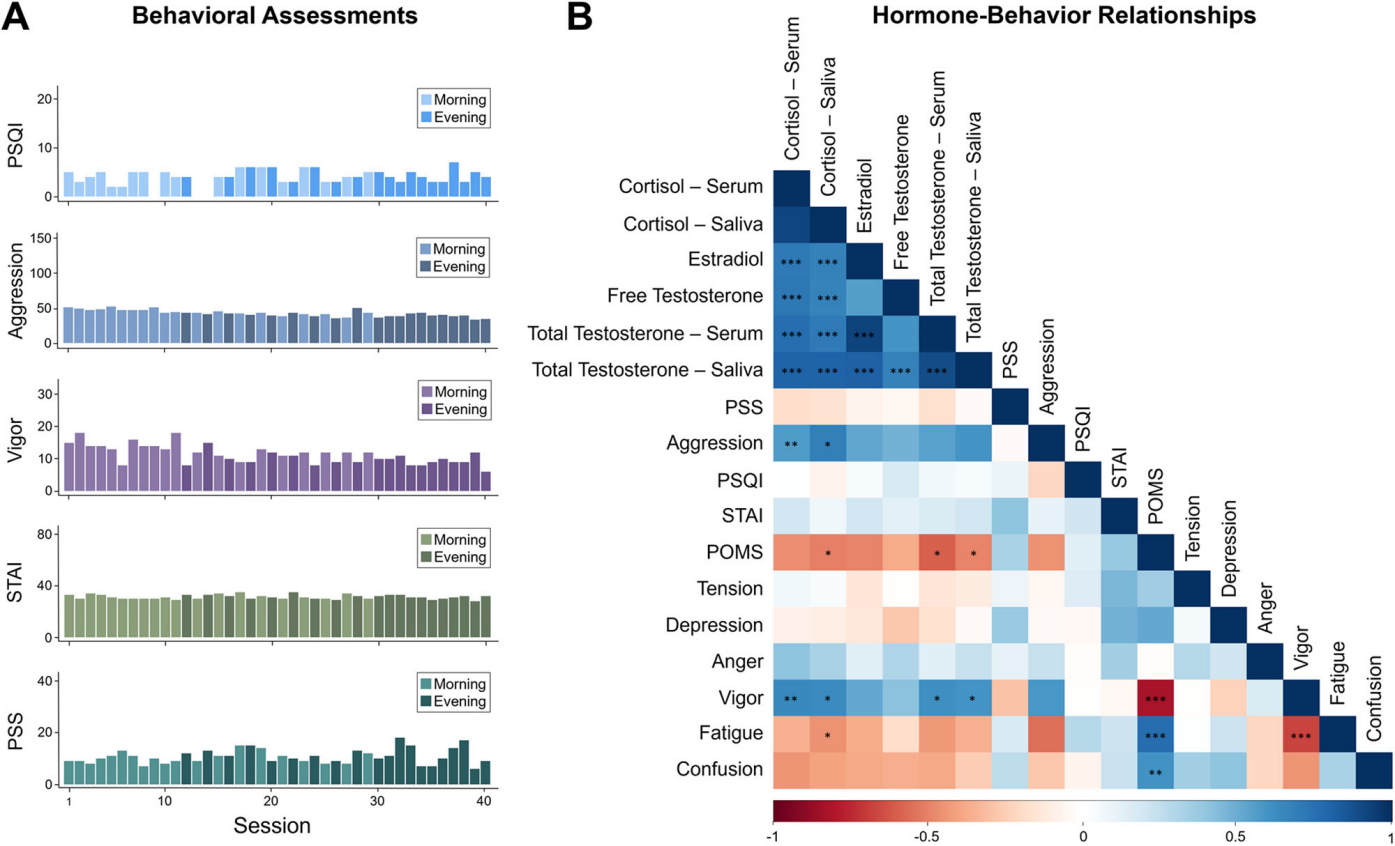
Behavioral variation across the 40-session experiment. ***A***, Measures of sleep, aggression, vigor, anxiety, and stress fall within standard ranges, with minimal variation across the study. Lighter colors indicate morning sessions and darker colors indicate evening sessions. ***B***, Correlation plot depicts relationships between steroid hormones and mood. Cool colors indicate positive correlations, warm colors indicate negative correlations. Asterisks indicate significant correlations after Bonferroni’s correction (*p < 0.00037; **p < 0.00007; ***p < 0.000007). Note correspondence between serum and salivary assessments of steroid hormones. Hormone correlations are reported in Extended Data Tables 2-1 through 2-3.

The following scales (adapted to reflect the past 12–24 h) were administered at each session (A.M. and P.M.): the Perceived Stress Scale (Cohen et al., 1983), State-Trait Anxiety Inventory for Adults (Speilberger and Vagg, 1984), Profile of Mood States (POMS; Pollock et al., 1979), and Aggression Questionnaire (Buss and Perry, 1992). The Pittsburgh Sleep Quality Index (Buysse et al., 1989) was acquired once per day to avoid redundancy. All mood measures fell within standard reference ranges. Indices of sleep quality, stress, and anxiety were unrelated to hormone values (Fig. 2). After correcting for multiple comparisons (Bonferroni corrected), cortisol concentrations were positively correlated with aggression (*r*_(38)_ = 0.54; *p *= 0.0003) and vigor (*r*_(38)_ = 0.58; *p *= 0.0001) and negatively correlated with overall POMS scores (*r*_(38)_ = −0.55; *p *= 0.0002) and fatigue (*r*_(38)_ = −0.54; *p *= 0.0003). Total testosterone concentrations were also positively correlated with vigor (*r*_(38)_ = 0.58; *p *= 0.0001) and negatively correlated with overall POMS scores (*r*_(38)_ = −0.58; *p *= 0.0001). Higher POMS scores indicate greater mood disturbance. No other hormone–behavior relationship met significance at a Bonferroni corrected threshold.

10.1523/JNEUROSCI.1856-23.2024.t2-1 Table 2-1 Correlations between gonadal hormones. Download Table 2-1, DOCX file.

10.1523/JNEUROSCI.1856-23.2024.t2-2 Table 2-2 Correlations between morning concentrations of gonadal hormones. Download Table 2-2, DOCX file.

10.1523/JNEUROSCI.1856-23.2024.t2-3 Table 2-3 Correlations between evening concentrations of gonadal hormones. Download Table 2-3, DOCX file.

### MRI acquisition

At each session, the participant underwent a structural MRI and 15 min eyes-open resting-state scan conducted on a Siemens 3 T Prisma scanner equipped with a 64-channel phased-array head coil. High-resolution anatomical scans were acquired using a T1-weighted magnetization prepared rapid gradient echo sequence (TR, 2,500 ms; TE, 2.31 ms; TI, 934 ms; flip angle, 7°; 0.8 mm thickness), followed by a gradient echo field map (TR, 758 ms; TE_1_, 4.92 ms; TE_2_, 7.38 ms; flip angle, 60°). Functional data were obtained via T2*-weighted multiband echo-planar imaging sequence sensitive to blood oxygenation level-dependent (BOLD) contrast (72 oblique slices; TR, 720 ms; TE, 37 ms; voxel size, 2 mm^3^; flip angle, 56°; MB factor, 8). In an effort to minimize motion, the head was secured with a 3D-printed foam head case.

### MRI preprocessing

#### Functional preprocessing

Initial preprocessing was performed using the Statistical Parametric Mapping 12 software (SPM12, Wellcome Trust Centre for Neuroimaging, London) in MATLAB. Functional data were realigned and unwarped to correct for head motion and geometric deformations due to motion and magnetic field inhomogeneities; the mean motion-corrected image was then coregistered to the high-resolution anatomical image. All scans were normalized to a subject-specific template using Advanced Normalization Tools’ (ANTs) multivariate template construction (Avants et al., 2011). A 4 mm full-width at half-maximum isotropic Gaussian kernel was subsequently applied to smooth the functional data. The following additional processing was performed using in-house MATLAB scripts. Global signal scaling (median, 1,000) was applied to account for fluctuations in signal intensity across space and time, and voxelwise timeseries were linearly detrended. Residual BOLD signal from each voxel was extracted after removing the effects of head motion and five physiological noise components (derived from CSF and white matter signal—although our use of coherence for functional connectivity allows for the estimation of frequency-specific covariances in spectral components below the range typically contaminated by physiological noise). Motion was modeled based on the Friston-24 approach, using a Volterra expansion of translational/rotational motion parameters, accounting for autoregressive and nonlinear effects of head motion on the BOLD signal (Friston et al., 1996). All nuisance regressors were detrended to match the BOLD timeseries. We note that steroid hormone concentrations were related to head motion (framewise displacement; FWD) due to generally greater movement during the evening sessions than morning sessions (*t*_(32.15)_ = −3.85; *p *< 0.001; *d *= 1.22). However, the average FWD was exceedingly minimal (*M *= 8 microns; SD* *= 4 microns), with a maximum of 930 microns across all 40 sessions. Nevertheless, to ensure this did not confound our findings, we specified a series of binary spike regressors for any frames that contained framewise displacements >500 microns (necessary in 17/40 sessions).

#### Functional connectivity estimation

Functional network nodes were defined by parcellating the brain based on the 400-region cortical (Schaefer et al., 2018) and 15 regions from the Harvard-Oxford subcortical atlas (http://www.fmrib.ox.ac.uk/fsl/). A summary timecourse for each session was extracted per node by taking the first eigenvariate across functional volumes (Friston et al., 2006). These regional timeseries were then decomposed into several frequency bands using a maximal overlap discrete wavelet transform. Low-frequency fluctuations in wavelets 3–6 (∼0.01–0.17 Hz) were selected for subsequent connectivity analyses (Patel and Bullmore, 2016). We estimated the spectral association between regional timeseries using magnitude-squared coherence: this yielded a 415 × 415 functional association matrix each day, whose elements indicated the strength of functional connectivity between all pairs of nodes (FDR-thresholded at *q *< 0.05). Coherence offers several advantages over alternative methods for assessing connectivity: (1) estimation of frequency-specific covariances, (2) simple interpretability (values are normalized to the [0,1] interval), and (3) robustness to temporal variability in hemodynamics between brain regions, which can otherwise introduce time-lag confounds to connectivity estimates via Pearson’s correlation.

### Statistical analyses

Statistical analyses were conducted in MATLAB (version R2022b) and R (version 4.3.2).

#### Estimating edgewise sensitivity to hormone fluctuations over time

First, we assessed time-synchronous variation in functional connectivity associated with testosterone, cortisol, and estradiol through a standardized regression analysis. Data were *Z*-transformed and edgewise coherence was regressed against hormonal timeseries to capture day-by-day variation in connectivity relative to hormonal fluctuations. For each model, we computed robust empirical null distributions of test statistics via 10,000 iterations of nonparametric permutation testing: under the null hypothesis of no temporal association between connectivity and hormones, the coherence data at each edge were randomly permuted, models were fit, and two-tailed *p* values were obtained as the proportion of models in which the absolute value of the permuted test statistics equaled or exceeded the absolute value of the “true” test statistics. We report edges surviving a threshold of *p *< 0.001. All edgewise regression maps were corrected for multiple comparisons (FDR-corrected at *q *< 0.05) before estimating mean nodal association strengths.

As an additional sensitivity test to account for variability in wakefulness between morning and evening sessions, we included time-since-waking at each session (i.e., ∼1 h since waking for morning sessions and ∼14 h since waking for evening sessions) as a regressor in a supplemental analysis.

#### Determining network sensitivity to hormone fluctuations

For each time-synchronous model, we examined the direction of hormone-related associations and whether particular networks were more or less sensitive to hormonal fluctuations. To that end, we took the thresholded statistical parametric maps for each model (where edges are test statistics quantifying the magnitude of association between coherence and hormonal timeseries) and estimated nodal association strengths per graph theory's treatment of signed, weighted networks. That is, positive and negative association strengths were computed independently for each of the 415 nodes by summing the suprathreshold positive/negative edges linked to them. We then assessed mean association strengths (±95% confidence intervals) in each direction across the various networks in our parcellation. We used the network parcellation used in Pritschet et al. (2020) for consistency across studies. Networks were defined by grouping the subnetworks of the 17-network Schaefer parcellation, such that the A, B, and C components of the Default Mode Network were treated as one network (Schaefer et al., 2018). The subcortical nodes of the Harvard-Oxford atlas were also treated as their own network, yielding a total of nine networks.

Finally, two-way ANOVAs with Tukey's HSD (*p *< 0.05, corrected for family-wise error) were used to compare the variance in nodal association strengths among the three hormones (testosterone, cortisol, and estradiol) and all nine functional networks. Additional ANOVAs were used to compare the variance in nodal association strengths associated with testosterone and estradiol fluctuations among both datasets (male and female) and all networks.

## Results

### Endocrine assessments

**Figure 3.**
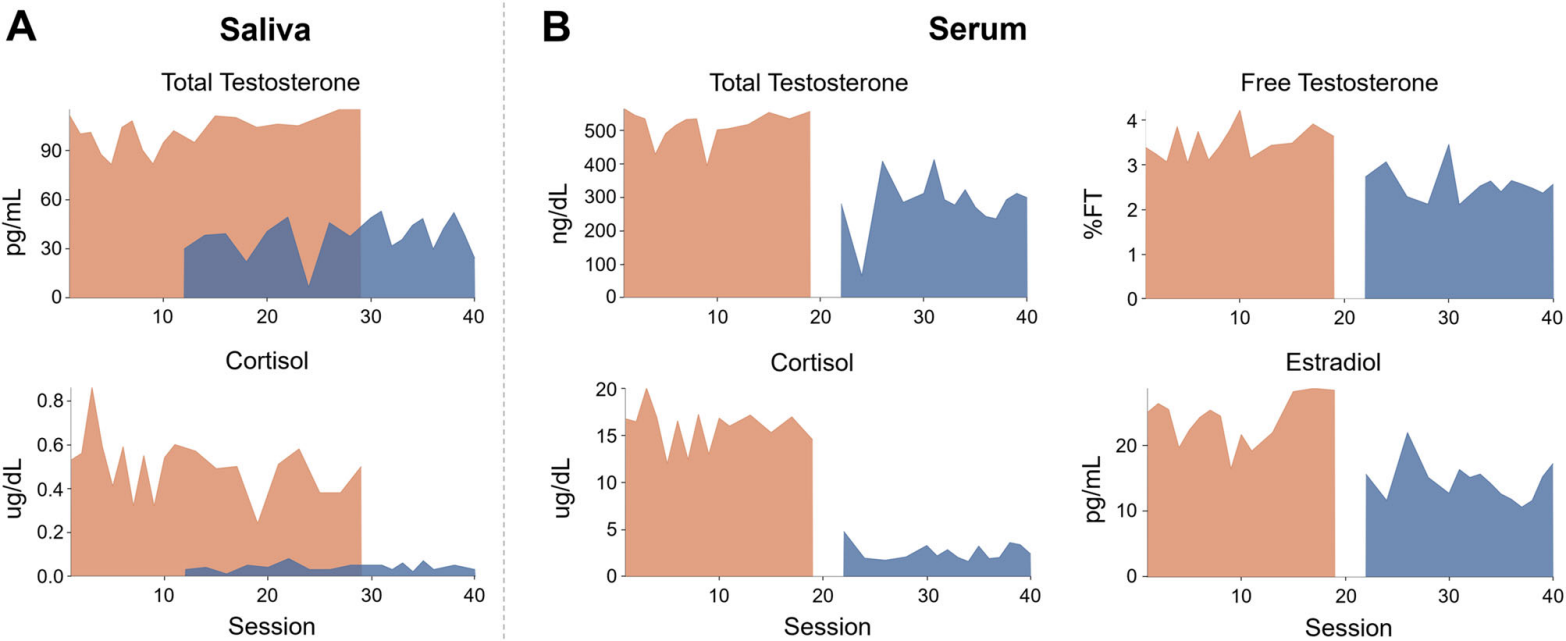
Steroid hormone concentrations by test session and time of day. ***A***, Saliva measurements for total testosterone and cortisol and ***B*** serum measurements for total testosterone, free testosterone, cortisol, and estradiol. All hormone concentrations were within or comparable to the standard range (Table 1). Note that saliva sampling was performed at each session; a blood sample was obtained once per day, following human subjects protocol regulations.

As expected, hormone concentrations peaked in the morning and dipped in the evening (Fig. 3, Table 1). Morning to evening decreases in testosterone, estradiol, and cortisol were ∼63, ∼39, and ∼92%, respectively. Testosterone, cortisol, and estradiol were correlated to each other, and hormone concentrations from saliva samples (testosterone and cortisol) were tightly correlated with their serum sample counterparts across the study (Fig. 2; Extended Data Table 2-1) and within morning-only and evening-only sessions (Extended Data Tables 2-2 and 2-3). Testosterone is generally available in two forms: either bound to a carrier protein (sex hormone-binding globulin) and therefore inert or bioavailable (free or loosely bound to albumin). For completeness, this dataset provides assessments of both free and total testosterone. To maximize available MRI sessions, analyses involving testosterone and cortisol reflect values derived from salivary samples obtained across all 40 sessions (Fig. 1). Analyses with serum concentrations are included in Extended Data Figure 4-2.

**Table 1.** Gonadal hormones by time of day

Hormone	Morning	Evening	Standard range	Units
	Mean (SD)	Mean (SD)		
Total testosterone (saliva)	101.6 (10.20)	37.9 (11.50)	41–141	pg/ml
Total testosterone (serum)	513.4 (47.00)	286.4 (79.00)	264–916	ng/dl
Free testosterone	3.5 (0.40)	2.6 (0.30)	1.5–3.2	%FT
Estradiol	23.8 (3.60)	14.5 (2.90)	7.6–42.6	pg/ml
Cortisol (saliva)	0.5 (0.10)	0.04 (0.02)	0.04–0.95	µg/dl
Cortisol (serum)	15.9 (2.10)	2.6 (0.90)	2.3–19.4	µg/dl

Standard reference ranges based on aggregate data from Labcorp (https://www.labcorp.com/) for the serum values and ZRT Lab (https://www.zrtlab.com/) for saliva values.

### Time-synchronous associations between steroid hormones and whole-brain functional connectivity

**Figure 4.**
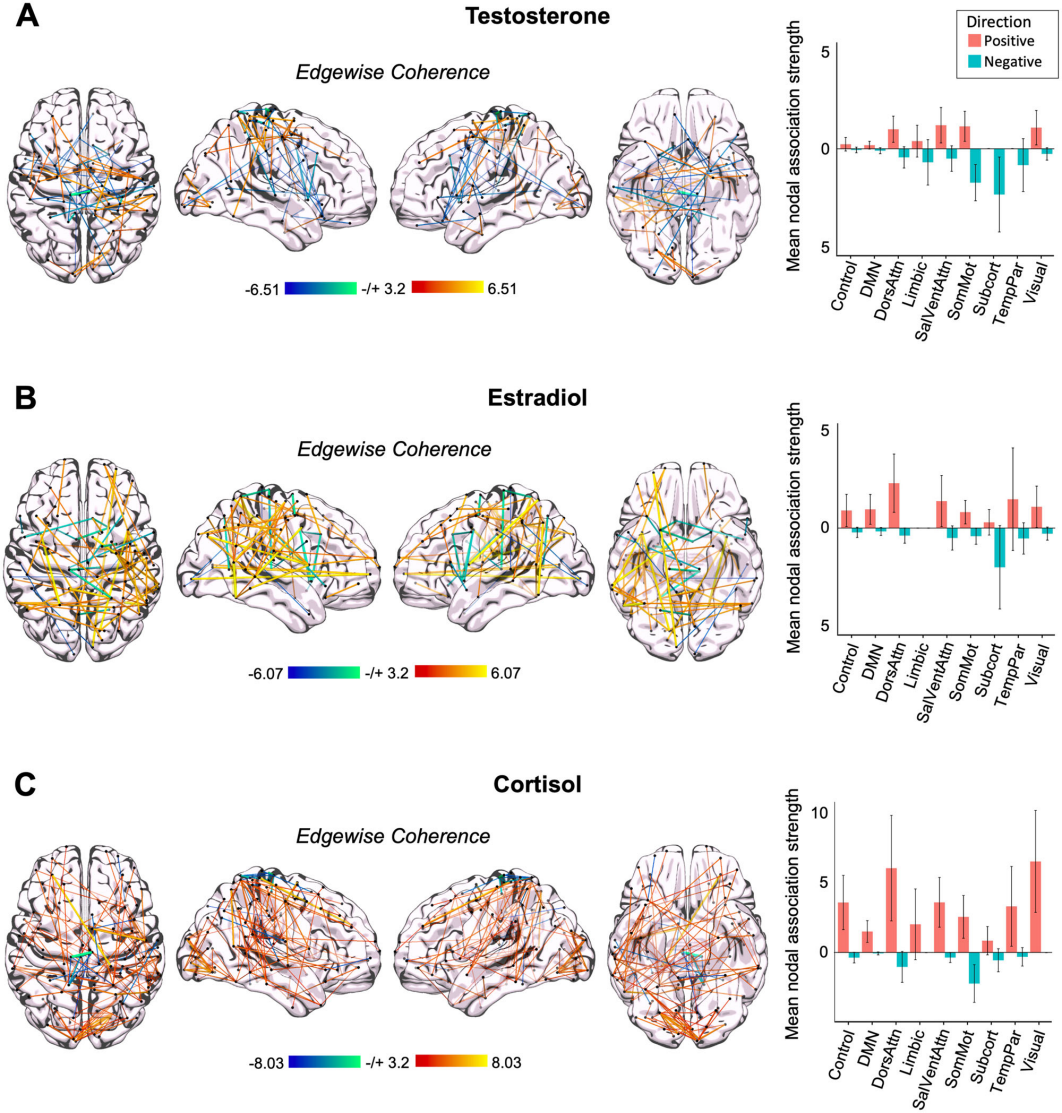
Whole-brain connectivity at rest is associated with intrinsic fluctuations in testosterone, cortisol, and estradiol in a densely sampled male. ***A***, Time-synchronous associations between total testosterone and coherence (left). Hotter colors indicate increased coherence with higher concentrations of testosterone; cool colors indicate the reverse. Reported edges survive a threshold of *p *< 0.001. Mean nodal association strengths (right). Error bars give 95% confidence intervals. All edges and mean nodal association strengths are corrected for multiple comparisons (FDR at *q *< 0.05). “Positive” refers to the average magnitude of positive associations (e.g., stronger coherence with higher testosterone concentrations); “Negative” refers to the average magnitude of inverse associations (e.g., weaker coherence with higher testosterone concentrations). DMN, Default Mode Network; DorsAttn, Dorsal Attention Network; SalVentAttn, Salience/Ventral Attention Network; SomMot, SomatoMotor Network; TempPar, Temporal Parietal Network. ***B***, Time-synchronous associations between estradiol and coherence (left) and mean nodal association strengths (right). ***C***, Time-synchronous associations between cortisol and coherence (left) and mean nodal association strengths (right). For additional analyses, see Extended Data Figures 4-1 through 4-6.

Previous work from our group (Pritschet et al., 2020) demonstrated robust increases in whole-brain coherence with increasing estradiol concentrations over the menstrual cycle in a naturally cycling female. Here, we tested the hypothesis that whole-brain resting-state functional connectivity in a male is associated with diurnal intrinsic fluctuations in total testosterone, cortisol, and estradiol in a time-synchronous (i.e., session-to-session) manner. Based on previous findings, we predicted that the Default Mode, Dorsal Attention, and Temporal Parietal networks would show the strongest associations with fluctuations in steroid hormone concentrations. Increases in cortisol were associated with heightened whole-brain functional connectivity across most of the nine functional networks analyzed (Fig. 4). Increases in testosterone and estradiol displayed mixed directions of mean nodal association strengths across functional networks. Notably, most networks showed some level of positive association strength with cortisol on average (with most 95% CIs not intersecting zero). Note that whole-brain connectivity strengths associated with fluctuations in free testosterone (Extended Data Fig. 4-1) were similar to total testosterone. Further, time-synchronous associations between serum concentrations of testosterone and cortisol were comparable with associations with salivary concentrations (though in the case of cortisol, serum associations were heightened relative to saliva; Extended Data Fig. 4-2). Global efficiency (a marker of within-network integration) did not differ significantly from morning to evening (*t*_(35.66)_ = 1.38; *p *= 0.177; *d *= 0.44), while participation (a marker of between-network integration) was heightened in the evening compared with that in the morning (*t*_(37.74)_ = −2.66; *p *= 0.011; *d *= −0.84; Extended Data Figs. 4-3 and 4-4). Next, to assess similarity in the spatial distribution of brain–hormone relationships (e.g., whether the same edges showed the same degree of sensitivity to testosterone and cortisol), we estimated the Spearman correlation between each pair of brain–hormone regression maps and found that brain–hormone associations are highly correlated between testosterone and cortisol, testosterone and estradiol, and cortisol and estradiol (*r*_(85,903)_ = 0.90, *p *< 0.001; *r*_(85,903)_ = 0.80, *p *< 0.001; *r*_(85,903)_ = 0.79, *p *< 0.001).

10.1523/JNEUROSCI.1856-23.2024.f4-1 Figure 4-1 **Whole-brain connectivity at rest is associated with intrinsic fluctuations in free testosterone.** Time-synchronous associations between free testosterone and coherence (top) and mean nodal association strengths (bottom). All edges and mean nodal association strengths are corrected for multiple comparisons (FDR at *q *< 0.05). Download Figure 4-1, TIF file.

10.1523/JNEUROSCI.1856-23.2024.f4-2 Figure 4-2 **Whole-brain connectivity associated with intrinsic fluctuations in testosterone and cortisol serum concentrations.** The magnitude of brain-testosterone relationships derived from serum values parallels what we see with testosterone saliva concentrations, but the magnitude of cortisol-brain associations derived from serum are greater than associations with cortisol saliva values. **(A)** Time-synchronous associations (top) and mean nodal association strengths (bottom) between total testosterone serum concentrations and coherence. **(B)** Time-synchronous associations (top) and mean nodal association strengths (bottom) between cortisol serum concentrations and coherence. All edges and mean nodal association strengths are corrected for multiple comparisons (FDR at *q *< 0.05). Download Figure 4-2, TIF file.

10.1523/JNEUROSCI.1856-23.2024.f4-3 Figure 4-3 **Efficiency values at each session by time of day.** Efficiency was not significantly different from morning to evening. Download Figure 4-3, TIF file.

10.1523/JNEUROSCI.1856-23.2024.f4-4 Figure 4-4 **Participation values at each session by time of day.** Participation was significantly greater in the evening than in the morning (*t*(37.74) = -2.66, *p *= .011, *d *= -0.84). Download Figure 4-4, TIF file.

**Figure 5.**
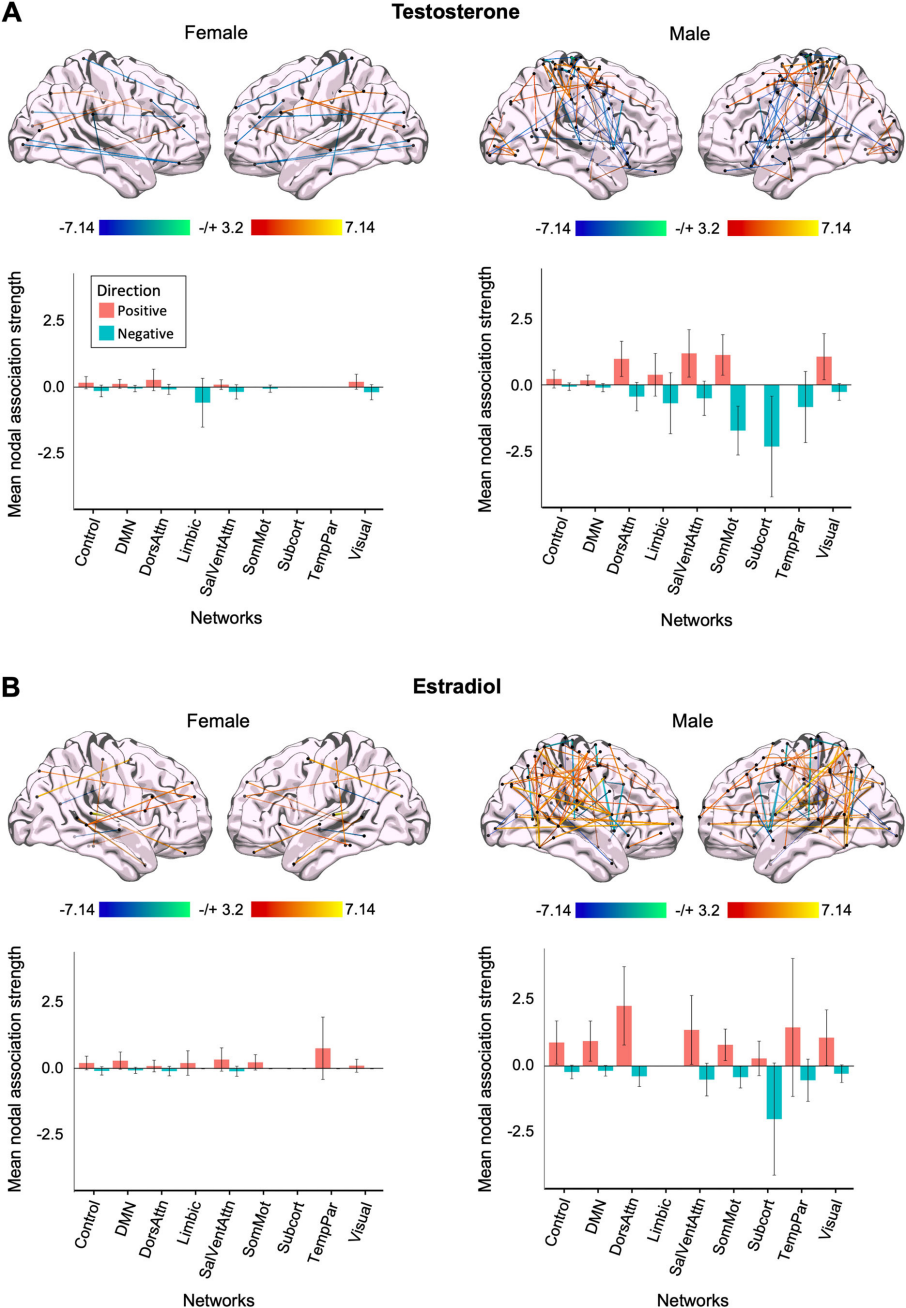
Intrinsic fluctuations in sex steroid hormones are associated with whole-brain resting-state functional connectivity in a male and a naturally cycling female. ***A***, Time-synchronous associations (top) and mean nodal association strengths (bottom) between total testosterone and coherence in a female (left) and male (right). ***B***, Time-synchronous associations (top) and mean nodal association strengths (bottom) between estradiol and coherence in a female (left) and male (right). Here, hormone–coherence relationships are exaggerated in the male. All edges and mean nodal association strengths are corrected for multiple comparisons (FDR at *q *< 0.05).

10.1523/JNEUROSCI.1856-23.2024.f4-5 Figure 4-5 **Time-synchronous brain-hormone associations accounting for awake time.** When incorporating time awake at each scan as a regressor, the average magnitude of brain-hormone associations differed in some networks, though overall trends remained. Time-synchronous associations between total testosterone **(A)**, estradiol **(B)**, and cortisol **(C)** and coherence, and mean nodal association strengths by network (bottom). All edges and mean nodal association strengths are corrected for multiple comparisons (FDR at *q *< 0.05). Download Figure 4-5, TIF file.

10.1523/JNEUROSCI.1856-23.2024.f4-6 Figure 4-6 **Morning and evening sessions show different patterns of whole-brain connectivity.** Steroid hormone concentrations show different patterns of whole-brain coherence and mean nodal association strengths in the morning sessions **(A)** compared to evening sessions **(B)**. Time-synchronous associations between total testosterone, estradiol, and cortisol and coherence (top), and mean nodal association strengths by network (bottom). All edges and mean nodal association strengths are corrected for multiple comparisons (FDR at *q *< 0.05). Download Figure 4-6, TIF file.

#### Positive associations between hormone concentrations and coherence differ across functional networks and hormones

Though trends in coherence across networks are somewhat overlapping across hormones, we wanted to further test differences in mean nodal association strengths by hormone and by network. To disentangle differences in coherence patterns positively associated with hormone concentrations, we used a two-way ANOVA to test the effects of each steroid hormone and functional network on positive brain–hormone associations. This revealed significant main effects of hormone (*F*_(2,1,218)_ = 34.606; *p *< 0.001) and network (*F*_(8,1,218)_ = 3.890; *p *< 0.001), but no significant interaction between hormones and networks (*F*_(16,1,218)_ = 1.581; *p *= 0.067), suggesting that brain–hormone relationships vary significantly by hormone as well as network but that trends across networks were not significantly different across hormones. To better understand the direction of these findings, we used Tukey's HSD post hoc analyses to reveal that cortisol–coherence associations were greater in magnitude than those observed for estradiol (*p *< 0.001) and testosterone (*p *< 0.001; Fig. 4*A*,*B*). Dorsal Attention and Visual networks displayed significantly greater hormone–brain associations, regardless of hormone, than the Default Mode Network (*p *= 0.001 and *p *= 0.008, respectively). Positive mean nodal association strengths in the Dorsal Attention Network were also significantly greater than those in the Limbic (*p *= 0.047) and Subcortical (*p *= 0.049) networks. Hormone–brain association strengths were also greater in the Visual Network than the Default Mode Network (*p *= 0.008).

As the participant's total wake time differed between morning (1 h) and evening (12–15 h) sessions, we accounted for this by including wake time into the models. General trends of whole-brain coherence remain the same across networks for all three hormones, though mean nodal association strengths are slightly diminished (Extended Data Fig. 4-5).

#### Negative associations between hormone concentrations and coherence differ across functional networks but not by hormone

To test the effects of hormone fluctuations and functional networks on negative mean nodal association strengths, we used a separate two-way ANOVA. This analysis uncovered a statistically significant main effect of functional network (*F*_(8,1,218)_ = 8.158; *p *< 0.001) but not hormone (*F*_(2,1,218)_ = 1.938; *p *= 0.144) and a significant interaction of hormones and networks (*F*_(16,1,218)_ = 2.022; *p *= 0.010). This implies that negative strengths differ across networks but not hormones, yet there is a different pattern of node strengths with each network for each hormone. For example, negative network associations with testosterone do not show similar magnitudes of associations with estradiol or cortisol fluctuations. Upon further investigation, Tukey's post hoc analyses revealed that across all hormones, the SomatoMotor and Subcortical networks showed strong negative mean nodal association strengths compared with most networks, including Control (*p *< 0.001 and *p *= 0.004, respectively), Default Mode (*p *< 0.001 and *p *< 0.001, respectively), Salience/Ventral Attention (*p *< 0.001 and *p *= 0.044, respectively), Limbic (*p *= 0.002 and *p *= 0.024, respectively), and Visual (*p *< 0.001 and *p *= 0.004, respectively) networks (Fig. 4). The SomatoMotor Network also displayed greater negative hormone–coherence associations than the Dorsal Attention Network (*p *= 0.011). Overall, the SomatoMotor and Subcortical networks showed significantly greater negative association strengths than other networks, though negative strengths in the SomatoMotor Network were most strongly associated with concentrations of cortisol and testosterone, but not estradiol, and negative strengths in the Subcortical Network were most strongly associated with concentrations of estradiol and testosterone.

### Whole-brain coherence tied to intrinsic fluctuations of sex hormones in both sexes

Then, to begin to understand the extent to which hormone–brain relationships are similar or different by sex, we compared whole-brain patterns of estradiol- and testosterone-related effects in our male participant to data from a companion dense-sampling study in a naturally cycling female (Fig. 5), collected under a near-identical protocol (Pritschet et al., 2020). Associations between sex hormones and whole-brain coherence were present across both sexes (Fig. 5*A*,*B*). In both participants, estradiol was associated with increased coherence in nearly all networks (with exception of the Subcortical Network in both participants and the SomatoMotor Network in the female participant). In contrast, testosterone exhibited varied associations for both participants. Notably, the magnitude of the relationship between endogenous hormone fluctuations and whole-brain coherence was heightened in the male participant (evident for both estradiol and testosterone). As a reference, the male participant experienced morning-to-evening decreases in testosterone, estradiol, and cortisol (∼63, ∼39, and ∼92%, respectively). The naturally cycling female participant experienced a 92% decrease in estradiol concentrations from ovulation to menses and a 40% decrease in testosterone concentrations from the peak during the luteal phase to menses. Next, we assessed the similarity in the spatial distribution of brain–hormone relationships (e.g., whether the same edges show sensitivity to testosterone and estradiol). We estimated the Spearman correlation between each pair of brain–hormone regression maps for each participant and found that brain–hormone associations were very weakly correlated between each participant and testosterone (*r*_(85,903)_ = 0.01; *p *= 0.002) and estradiol (*r*_(85,903)_ = −0.04; *p *< 0.001). Within the female participant, brain–hormone associations between testosterone and estradiol were also very weakly correlated (*r*_(85,903)_ = −0.02; *p *< 0.001).

#### Testosterone relationships with functional connectivity differ by sex and network

To directly compare differences in mean node strengths positively associated with testosterone concentrations in both participants, we ran a two-way ANOVA to compare the main effects of sex and functional network on positive association strengths. This revealed a statistically significant main effect of network (*F*_(8,812)_ = 2.098; *p *= 0.034) and sex (*F*_(1,812)_ = 22.567; *p *< 0.001), and a significant interaction between sex and network (*F*_(8,812)_ = 1.970; *p *= 0.047), on positive mean nodal association strengths of whole-brain coherence associated with total testosterone fluctuations. Positive mean nodal association strengths were significantly greater in the male participant. No comparisons of positive strengths across networks survive Tukey's post hoc analyses. In the female participant, the strongest associations with testosterone were in the Dorsal Attention, Visual, and Control networks. In the male participant, the strongest associations with testosterone were in the Salience/Ventral Attention, SomatoMotor, Dorsal Attention, and Visual Networks (Fig. 5*A*).

We ran a subsequent two-way ANOVA to test the impact of sex and functional network on negative mean nodal association strengths with estradiol and found a main effect of sex (*F*_(1,812)_ = 50.73; *p *< 0.001) and network (*F*_(8,812)_ = 10.83; *p *< 0.001), as well as an interaction between sex and network (*F*_(8,812)_ = 12.57; *p *< 0.001). Overall, negative strengths associated with testosterone concentrations were greater in the male than the female participant. The SomatoMotor and Subcortical networks displayed the strongest negative associations with fluctuations in testosterone and were significantly greater than the Control (*p *= 0.004 and *p *= 0.046, respectively) and Default Mode (*p *< 0.001 and *p *= 0.028, respectively) networks. Both networks were also significantly greater in the male than the female participant (*p *< 0.001 and *p *= 0.014, respectively).

#### Associations between estradiol concentrations and coherence are significantly greater in the male participant

To examine differences in positive associations with estradiol, we ran a two-way ANOVA to compare the main effects of sex and functional network on positive association strengths. This uncovered a statistically significant main effect of sex (*F*_(1,812)_ = 21.316; *p *< 0.001), but not network (*F*_(8,812)_ = 1.199; *p *= 0.296), on positive mean nodal association strengths of whole-brain coherence associated with estradiol fluctuations, such that the male participant exhibited stronger positive associations between estradiol fluctuations and coherence. This is notable given the significant difference in estradiol concentrations between both participants (*t*_(29.64)_ = −6.39; *p *< 0.001; *d *= 1.65). Additionally, there was no significant interaction between sex and network (*F*_(8,812)_ = 1.219; *p *= 0.285). No comparisons of positive strengths across networks survive Tukey's post hoc analyses. When comparing equivalent networks in both sexes, the Dorsal Attention Network was significantly greater in the male participant (*p *= 0.004).

When examining the effects of sex and network on negative mean nodal association strengths tied to estradiol concentrations, we observed a main effect of sex (*F*_(1,812)_ = 17.886; *p *< 0.001) and network (*F*_(8,812)_ = 2.468; *p *= 0.012), as well as a significant interaction between sex and network (*F*_(8,812)_ = 2.653; *p *= 0.007). Overall, negative strengths associated with estradiol concentrations were greater in the male than those in the female participant. Further, Tukey's HSD post hoc analyses identified that the Subcortical Network displayed significantly greater negative strengths than the Control (*p *= 0.005), Default Mode (*p *= 0.002), Dorsal Attention (*p *= 0.025), Limbic (*p *= 0.004), SomatoMotor (*p *= 0.011), and Visual Networks (*p *= 0.007). Negative strengths within the Subcortical Network were significantly greater in the male than the female participant (*p *< 0.001).

#### Nodes most strongly tied to fluctuation in steroid hormones differ by sex

To determine similarities in brain–hormone associations at the level of individual nodes, we compared the strongest mean nodal association strengths across participants. Within the male participant, nodes in the Dorsal Attention, Salience/Ventral Attention, and Visual Networks demonstrated the strongest association strengths with diurnal variation among/across all three steroid hormones. Nodes in the right postcentral region (Dorsal Attention Network), right extrastriate cortex (Visual Network), and right somatomotor regions showed the greatest positive associations between functional activation and diurnal variation in all three steroid hormones. Further, right temporal pole (Limbic Network) and left lateral prefrontal cortex (PFC; Ventral Attention Network) regions were strongly tied to fluctuations in testosterone and cortisol concentrations, but not to fluctuations in estradiol. Conversely, right temporal lobe regions specifically belonging to the Control Network were most strongly tied to diurnal fluctuations of estradiol and cortisol concentrations. These associations were also present within right parietal occipital and superior parietal lobes (Dorsal Attention Network).

Nodes most strongly negatively associated with concentrations of testosterone and cortisol included bilateral somatomotor regions, left postcentral regions (Dorsal Attention Network), left orbitofrontal cortex (Limbic Network), and right temporal parietal regions. Negative associations with estradiol were observed in the left frontal medial region and right medial posterior PFC (Ventral Attention Network) and left somatomotor regions.

The nodes tied most strongly to estradiol fluctuations in the female participant included the right temporal lobe (Default Mode Network) and right lateral PFC (Ventral Attention Network), while the left precentral ventral region (Dorsal Attention Network) was most strongly tied to fluctuations in testosterone.

## Discussion

This precision imaging study reveals that rhythmic changes in the brain's functional network architecture are tied to diurnal fluctuations in steroid hormones. In a previous study, we mapped the brain's response to intrinsic fluctuations in sex hormones across the menstrual cycle in a densely sampled female (Fitzgerald et al., 2020; Taylor et al., 2020; Mueller et al., 2021; Pritschet et al., 2020, 2021). Here, we extend those findings by densely sampling a male who underwent (f)MRI scanning and venipuncture every 12–24 h, capturing the brain's response to diurnal changes in steroid hormone secretion from morning to night. Fluctuations in testosterone, cortisol, and estradiol were tied to changes in functional coherence across most of the cortex. In particular, functional connectivity within the Dorsal Attention Network demonstrated the strongest relationships across all three hormones. Comparisons between the densely sampled male and female revealed that testosterone and estradiol fluctuations influence intrinsic network properties in both sexes with exaggerated effects in the male, demonstrating that sex hormones impact brain function in both sexes and these effects are not limited to the menstrual cycle.

Existing evidence supports an association between time-of-day and functional connectivity. Sparse sampling studies investigating the impact of diurnal variation on brain dynamics tested subjects at 2+ timepoints (e.g., morning, afternoon, and/or evening sessions) and compared brain metrics across these timepoints, revealing a time-of-day effect on various measures of network topology (Marek et al., 2010; Shannon et al., 2013; Orban et al., 2020; Farahani et al., 2022). Consistent with our findings, Farahani et al. (2022) found that the Ventral Attention and Visual networks exhibit decreased functional connectivity from morning to evening sessions, while the SomatoMotor Network was more active in evening sessions. Our dense-sampling study, which tracks dynamic changes in the brain over time with high temporal precision, sheds further light on these findings by probing a specific biological driver of time-of-day effects in network connectivity: namely, diurnal steroid hormone fluctuations. Analysis of brain–hormone relationships within morning-only and evening-only sessions reveals differential patterns of sensitivity to these factors (Extended Data Fig. 4-6), a motivating factor to keep time-of-day consistent within MRI studies.

Though we cannot test causal mechanisms underlying how steroid hormones influence network dynamics, we know from rodent models that steroid hormones induce rapid effects on cell morphology. In rats, hormone-induced changes in synaptic plasticity have been studied extensively in hippocampal CA1 neurons, where fluctuations in androgen and estradiol concentrations are correlated with dendritic spine density (Leranth et al., 2003, 2004; MacLusky et al., 2006; Hojo et al., 2008; Li et al., 2012; Tozzi et al., 2019). Our data suggest that rapid effects of steroid hormones are also observable at the macroscopic level of intrinsic functional networks in the human brain. The AM diurnal peak in steroid hormones is tied to heightened whole-brain coherence, particularly in the Dorsal Attention, Salience/Ventral Attention, and Visual Networks.

Direct comparisons between a male and female participant reveal that intrinsic fluctuations in steroid hormones—be it across the menstrual cycle or diurnal cycle—modulate cortical activity in the human brain and the magnitude of these effects are greater in the male participant. Dynamic changes in estradiol production impacted brain networks to a greater degree in a densely sampled male than a naturally cycling female. We see this effect despite a significant sex difference in estradiol concentrations. Further, diurnal changes in testosterone had a greater impact on brain networks in the male participant than those observed in the female participant across the cycle. Note that testosterone is aromatized into estradiol, which could contribute to the observed effects on brain network dynamics. Importantly, these data challenge the erroneous notion that estradiol and testosterone are female- and male-specific hormones—here, we demonstrate that both hormones are associated with dynamic changes in the brain's functional architecture, regardless of sex.

Diurnal rhythms are ubiquitous across mammalian species and an organizing principle for human physiology and behavior. This precision imaging study sheds light on the extent to which intrinsic rhythms in hormone production influence large-scale brain networks by collecting fluid biomarkers and MRI data on a participant at an unprecedented temporal frequency. One limiting factor of single subject precision imaging studies is the extent to which the observed findings generalize to the broader population. Behavioral factors that vary by time of day, including sleep quality, stress, and aggression, may contribute to the observed changes in brain network dynamics. However, it is unlikely that these factors could fully explain our observed results given that the participant maintained a consistent and low level of stress and aggression and maintained a stable sleep schedule throughout the duration of the study. Future dense-sampling studies could expand on our results by investigating the extent to which these observations are universal versus idiosyncratic across a diverse range of individuals. The magnitude of diurnal variation in hormone concentrations diminishes with advanced age, so variation in whole-brain coherence from morning to evening may weaken in parallel—an important topic for future investigation.

Other physiological factors such as alertness, respiratory rate, global changes in blood flow, or other endocrine changes such as melatonin could also contribute to the observed results. Orban et al. (2020) found that the magnitude of fluctuations in global brain activity decline throughout the day. When factoring in global activity into our analysis, we observed an overall decrease in mean nodal association strengths despite a stable overall pattern of strengths across networks, suggesting that global activity can account for only some of the diurnal variation within networks. Moreover, melatonin production and individual sleep–wake preference (i.e., individuals with earlier vs later sleep and wake times) has been shown to affect functional connectivity within the Default Mode Network, with “night owl” subjects displaying reduced functional connectivity during typical work hours (Facer-Childs et al., 2019). While our precision imaging study avoided these confounds (the subject displayed a highly metronomic sleep/wake cycle over the 40 d experiment), future studies examining diurnal rhythms in an expanded sample should control for individual sleep–wake preferences. In a supplemental analysis, we included total awake time at each session as a regressor to control for potential differences due to alertness (Extended Data Fig. 4-5), and the pattern of results did not change appreciably.

This dataset provides a novel approach for understanding endocrine modulation of functional networks and provides a roadmap for future studies to probe the functional significance of these rhythms for behavior. Combined with our findings in a naturally cycling female, these results further demonstrate steroid hormones’ ability to modulate brain function across both sexes, and further research in this field is critical for expanding our basic understanding of endocrine influences on the brain.

## Data Availability

The dataset consists of 40 MRI scans (T1w, T2w, and resting-state functional scans) alongside state-dependent measures and serum assessments of steroid hormones for each session. The data will be publicly available on https://openneuro.org/ upon publication.
