# Supplementary material for: Diurnal Fluctuations in Steroid Hormones Tied to Variation in Intrinsic Functional Connectivity in a Densely Sampled Male
Source: J Neurosci. 2024 Apr 16;44(22):e1856232024. doi: 10.1523/JNEUROSCI.1856-23.2024 (PMC11140665; doi:10.1523/JNEUROSCI.1856-23.2024)
Supplement: Table 2-1 — Correlations between gonadal hormones. Download Table 2-1, DOCX file. [file jneuro-44-e1856232024-s001.docx]

| Hormone pair | | t-statistic | p-value | Pearson’s correlation |
| --- | --- | --- | --- | --- |
| Cortisol (saliva) | Cortisol (serum) | 19.98 | 2.2e-16*** | 0.97 |
| Cortisol (saliva) | Total Testosterone (saliva) | 11.38 | 8.4e-14*** | 0.88 |
| Cortisol (saliva) | Total Testosterone (serum) | 7.45 | 4.1e-08*** | 0.82 |
| Cortisol (saliva) | Estradiol | 6.05 | 1.6e-06*** | 0.75 |
| Cortisol (saliva) | Free Testosterone | 5.49 | 7.3e-06*** | 0.72 |
| Total Testosterone (saliva) | Total Testosterone (serum) | 15.22 | 4.6e-15*** | 0.94 |
| Total Testosterone (saliva) | Cortisol (serum) | 13.90 | 4.3e-14*** | 0.93 |
| Total Testosterone (saliva) | Estradiol | 8.88 | 1.2e-09*** | 0.86 |
| Total Testosterone (saliva) | Free Testosterone | 5.65 | 4.7e-06*** | 0.73 |
| Cortisol (serum) | Total Testosterone (serum) | 9.24 | 5.3e-10*** | 0.87 |
| Cortisol (serum) | Estradiol | 7.57 | 3.0e-08*** | 0.82 |
| Cortisol (serum) | Free Testosterone | 7.31 | 5.8e-08*** | 0.81 |
| Total Testosterone (serum) | Estradiol | 11.83 | 2.1e-12*** | 0.91 |
| Total Testosterone (serum) | Free Testosterone | 3.92 | 5.2e-04** | 0.60 |
| Estradiol | Free Testosterone | 3.87 | 6.0e-04** | 0.59 |
| Note. Bonferroni adjusted alpha for 15 comparisons: *p<0.003, **p<0.0007, ***p<0.00007 | | | | |
