# Supplementary material for: Diurnal Fluctuations in Steroid Hormones Tied to Variation in Intrinsic Functional Connectivity in a Densely Sampled Male
Source: J Neurosci. 2024 Apr 16;44(22):e1856232024. doi: 10.1523/JNEUROSCI.1856-23.2024 (PMC11140665; doi:10.1523/JNEUROSCI.1856-23.2024)
Supplement: Table 2-2 — Correlations between morning concentrations of gonadal hormones. Download Table 2-2, DOCX file. [file jneuro-44-e1856232024-s002.docx]

| Hormone pair | | t-statistic | p-value | Pearson’s correlation |
| --- | --- | --- | --- | --- |
| Cortisol (saliva) | Cortisol (serum) | 5.92 | 5.1e-05*** | 0.85 |
| Cortisol (saliva) | Total Testosterone (saliva) | -0.19 | 0.86 | -0.04 |
| Cortisol (saliva) | Total Testosterone (serum) | 0.44 | 0.66 | 0.12 |
| Cortisol (saliva) | Estradiol | -0.12 | 0.91 | -0.03 |
| Cortisol (saliva) | Free Testosterone | -0.53 | 0.60 | -0.15 |
| Total Testosterone (saliva) | Total Testosterone (serum) | 4.30 | 8.7e-04* | 0.77 |
| Total Testosterone (saliva) | Cortisol (serum) | 0.95 | 0.36 | 0.25 |
| Total Testosterone (saliva) | Estradiol | 3.75 | 2.4e-03* | 0.72 |
| Total Testosterone (saliva) | Free Testosterone | -0.24 | 0.82 | -0.07 |
| Cortisol (serum) | Total Testosterone (serum) | 1.07 | 0.31 | 0.28 |
| Cortisol (serum) | Estradiol | 0.68 | 0.51 | 0.18 |
| Cortisol (serum) | Free Testosterone | 0.55 | 0.59 | 0.15 |
| Total Testosterone (serum) | Estradiol | 5.94 | 5.0e-05*** | 0.86 |
| Total Testosterone (serum) | Free Testosterone | -1.24 | 0.24 | -0.32 |
| Estradiol | Free Testosterone | -0.42 | 0.68 | -0.12 |
| Note. Bonferroni adjusted alpha for 15 comparisons: *p<0.003, **p<0.0007, ***p<0.00007 | | | | |
