# Supplementary material for: Diurnal Fluctuations in Steroid Hormones Tied to Variation in Intrinsic Functional Connectivity in a Densely Sampled Male
Source: J Neurosci. 2024 Apr 16;44(22):e1856232024. doi: 10.1523/JNEUROSCI.1856-23.2024 (PMC11140665; doi:10.1523/JNEUROSCI.1856-23.2024)
Supplement: Table 2-3 — Correlations between evening concentrations of gonadal hormones. Download Table 2-3, DOCX file. [file jneuro-44-e1856232024-s003.docx]

| Hormone pair | | t-statistic | p-value | Pearson’s correlation |
| --- | --- | --- | --- | --- |
| Cortisol (saliva) | Cortisol (serum) | 3.41 | 4.7e-03 | 0.69 |
| Cortisol (saliva) | Total Testosterone (saliva) | 1.76 | 0.10 | 0.38 |
| Cortisol (saliva) | Total Testosterone (serum) | 0.22 | 0.83 | 0.06 |
| Cortisol (saliva) | Estradiol | -0.31 | 0.76 | -0.09 |
| Cortisol (saliva) | Free Testosterone | -0.07 | 0.95 | -0.02 |
| Total Testosterone (saliva) | Total Testosterone (serum) | 3.91 | 1.8e-03* | 0.74 |
| Total Testosterone (saliva) | Cortisol (serum) | 1.53 | 0.15 | 0.39 |
| Total Testosterone (saliva) | Estradiol | 0.50 | 0.62 | 0.14 |
| Total Testosterone (saliva) | Free Testosterone | -0.89 | 0.39 | -0.24 |
| Cortisol (serum) | Total Testosterone (serum) | 0.13 | 0.90 | 0.04 |
| Cortisol (serum) | Estradiol | -0.47 | 0.65 | -0.13 |
| Cortisol (serum) | Free Testosterone | 0.66 | 0.52 | 0.18 |
| Total Testosterone (serum) | Estradiol | 3.12 | 8.1e-03 | 0.66 |
| Total Testosterone (serum) | Free Testosterone | -1.97 | 0.07 | -0.48 |
| Estradiol | Free Testosterone | -1.72 | 0.11 | -0.43 |
| Note. Bonferroni adjusted alpha for 15 comparisons: *p<0.003, **p<0.0007, ***p<0.00007 | | | | |
