# Supplementary figures and images for: Diurnal Fluctuations in Steroid Hormones Tied to Variation in Intrinsic Functional Connectivity in a Densely Sampled Male
Source: J Neurosci. 2024 Apr 16;44(22):e1856232024. doi: 10.1523/JNEUROSCI.1856-23.2024 (PMC11140665; doi:10.1523/JNEUROSCI.1856-23.2024)

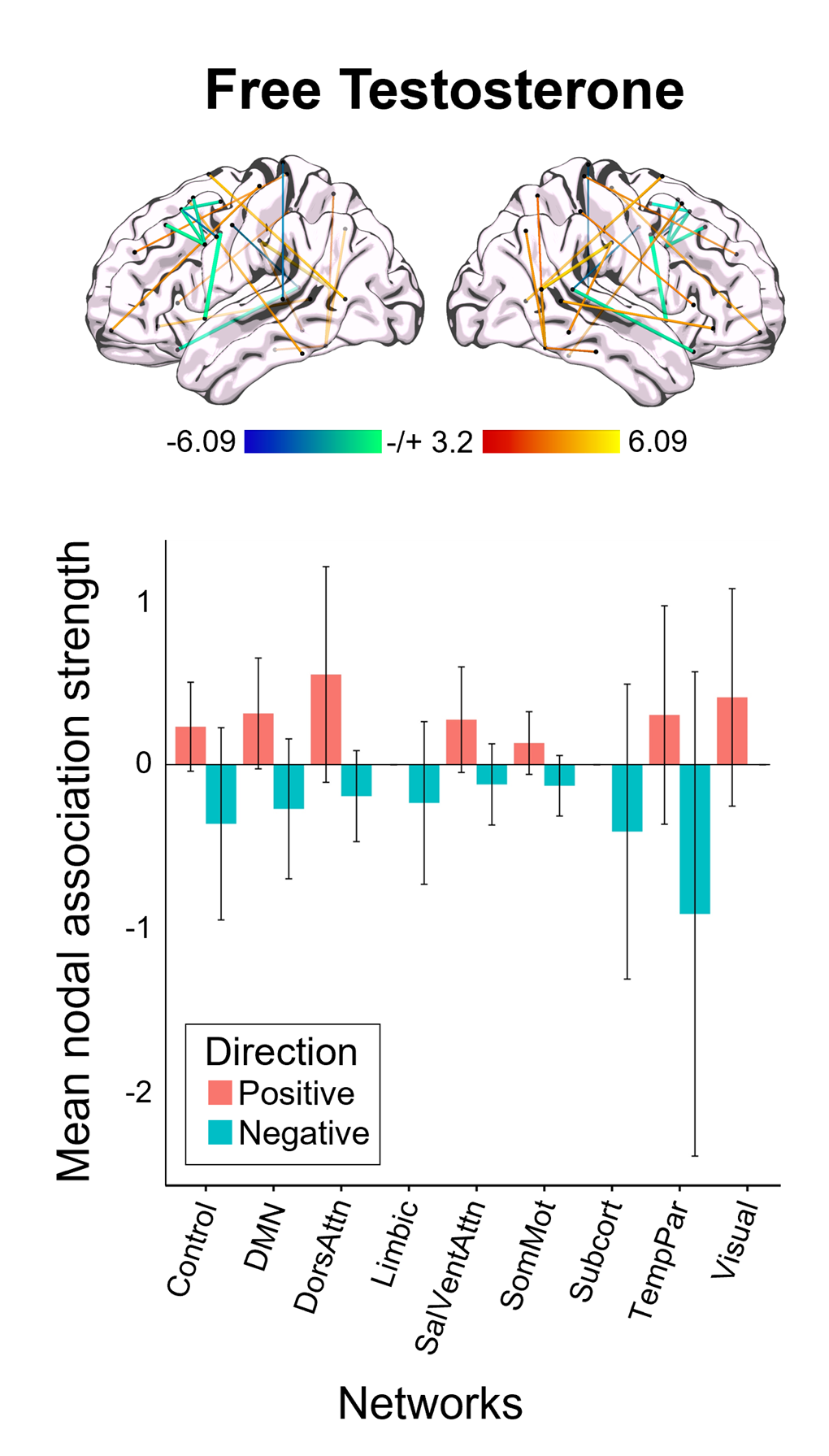

Supplement: Figure 4-1 — Whole-brain connectivity at rest is associated with intrinsic fluctuations in free testosterone. Time-synchronous associations between free testosterone and coherence (top) and mean nodal association strengths (bottom). All edges and mean nodal association strengths are corrected for multiple comparisons (FDR at q < 0.05). Download Figure 4-1, TIF file. [file jneuro-44-e1856232024-s004.tif]

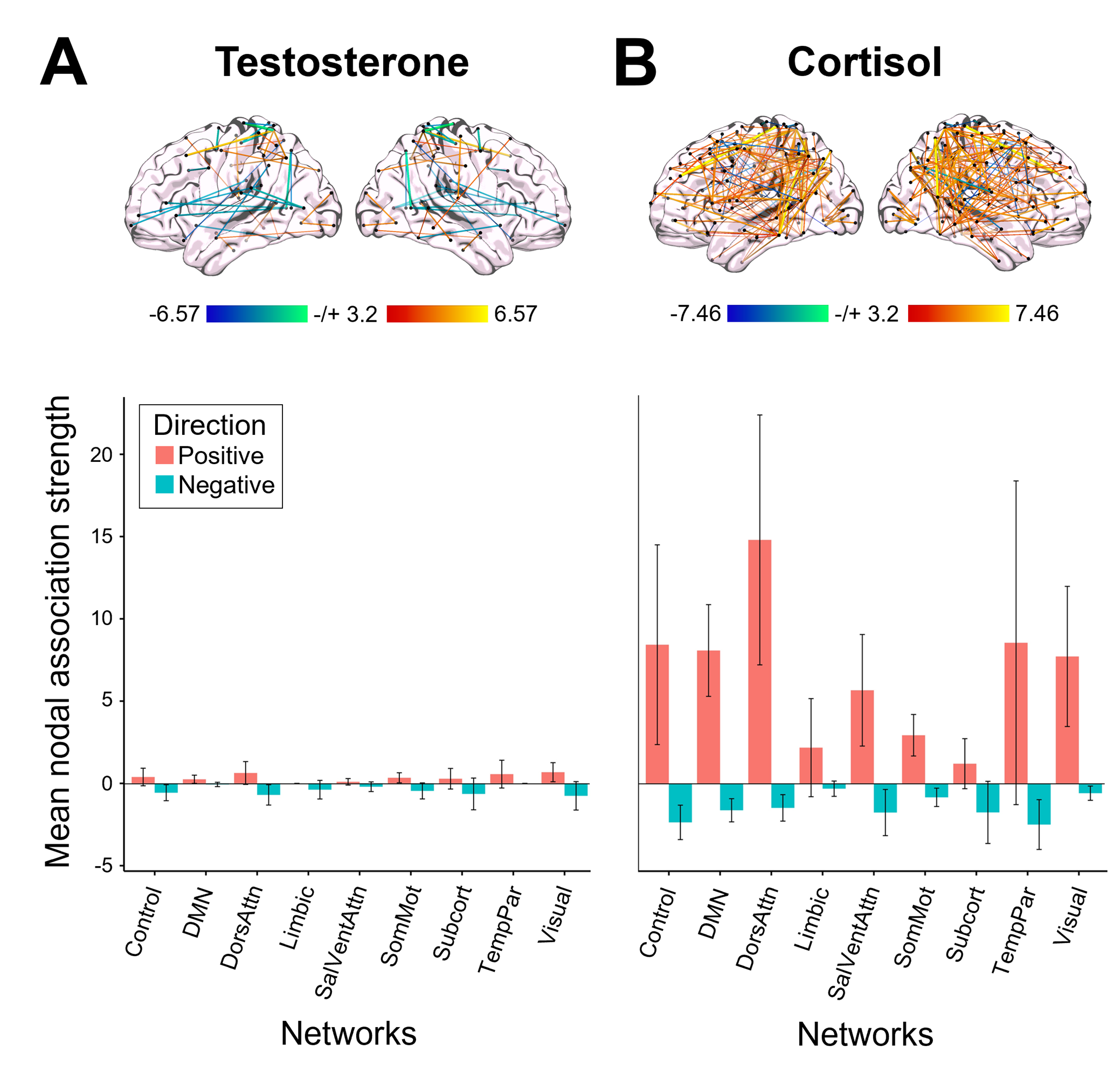

Supplement: Figure 4-2 — Whole-brain connectivity associated with intrinsic fluctuations in testosterone and cortisol serum concentrations. The magnitude of brain-testosterone relationships derived from serum values parallels what we see with testosterone saliva concentrations, but the magnitude of cortisol-brain associations derived from serum are greater than associations with cortisol saliva values. (A) Time-synchronous associations (top) and mean nodal association strengths (bottom) between total testosterone serum concentrations and coherence. (B) Time-synchronous associations (top) and mean nodal association strengths (bottom) between cortisol serum concentrations and coherence. All edges and mean nodal association strengths are corrected for multiple comparisons (FDR at q < 0.05). Download Figure 4-2, TIF file. [file jneuro-44-e1856232024-s005.tif]

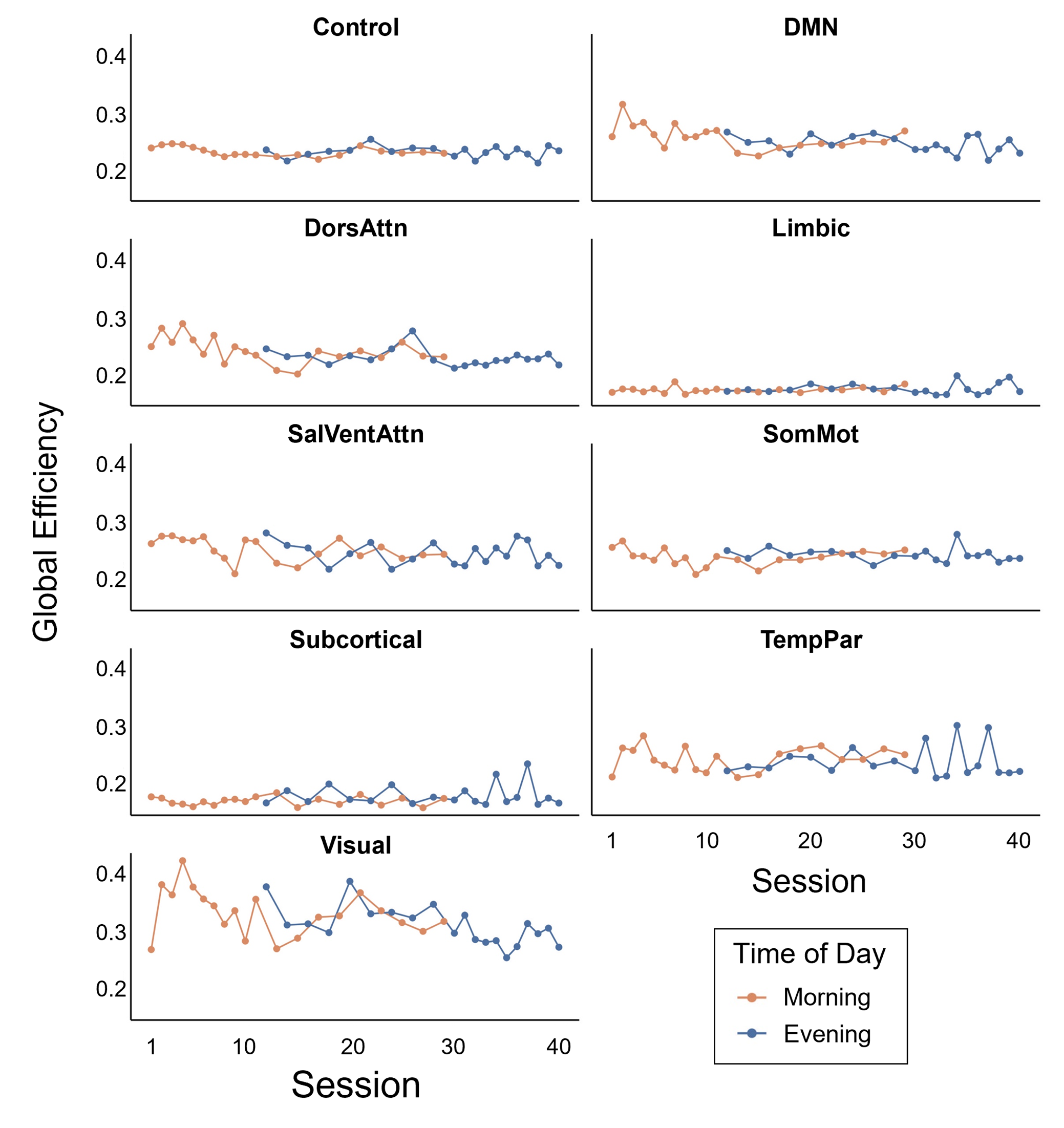

Supplement: Figure 4-3 — Efficiency values at each session by time of day. Efficiency was not significantly different from morning to evening. Download Figure 4-3, TIF file. [file jneuro-44-e1856232024-s006.tif]

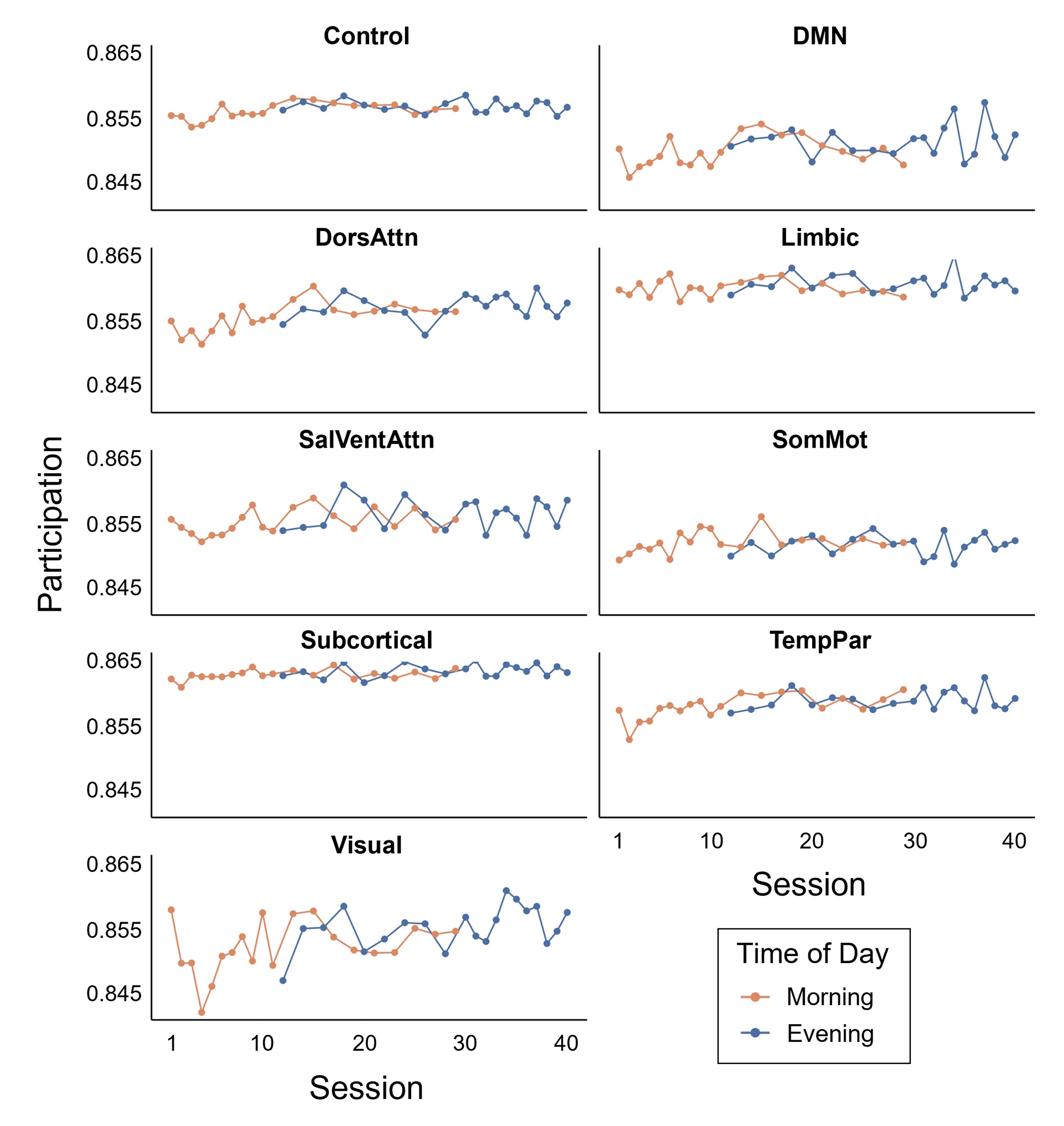

Supplement: Figure 4-4 — Participation values at each session by time of day. Participation was significantly greater in the evening than in the morning (t(37.74) = -2.66, p = .011, d = -0.84). Download Figure 4-4, TIF file. [file jneuro-44-e1856232024-s007.tif]

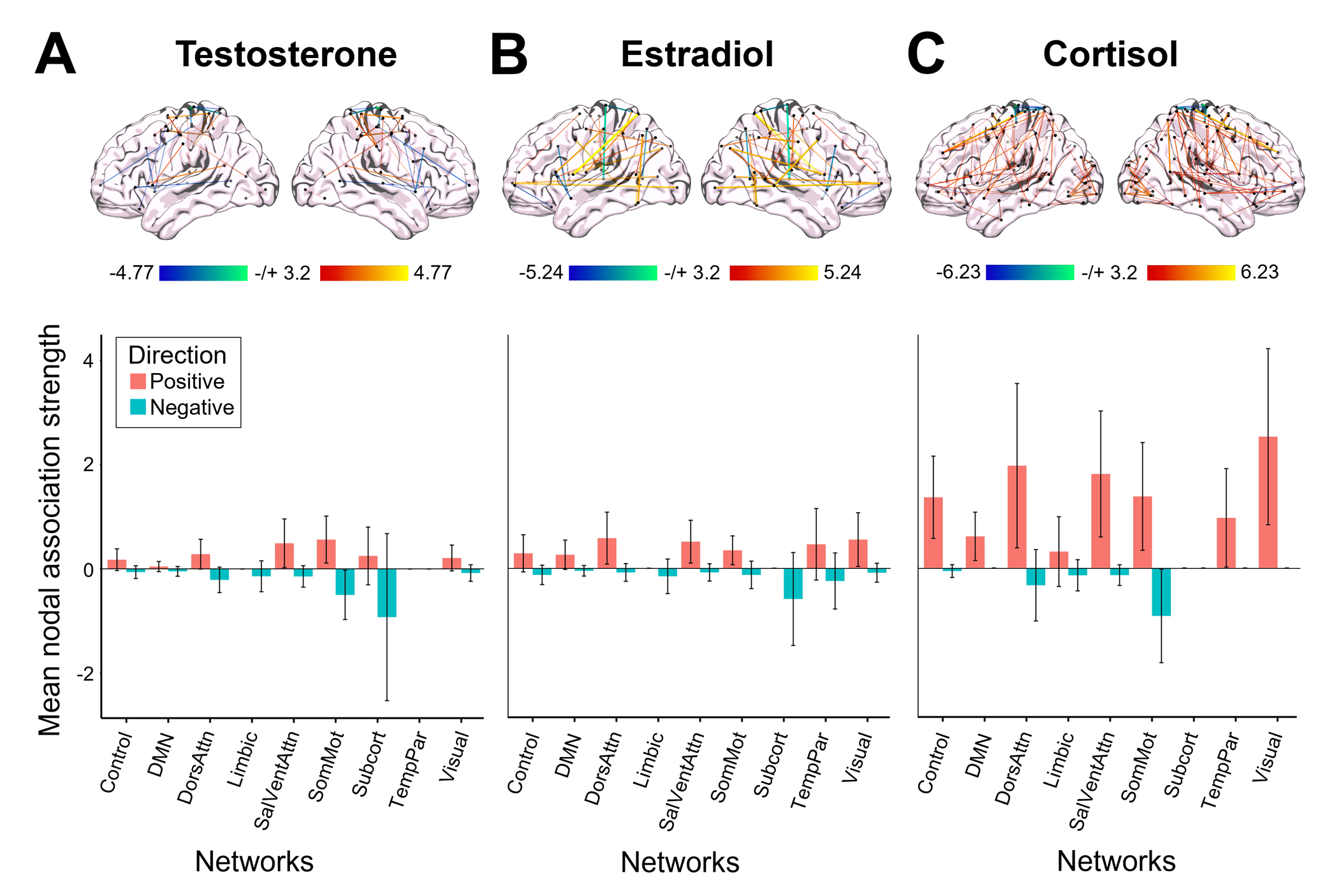

Supplement: Figure 4-5 — Time-synchronous brain-hormone associations accounting for awake time. When incorporating time awake at each scan as a regressor, the average magnitude of brain-hormone associations differed in some networks, though overall trends remained. Time-synchronous associations between total testosterone (A), estradiol (B), and cortisol (C) and coherence, and mean nodal association strengths by network (bottom). All edges and mean nodal association strengths are corrected for multiple comparisons (FDR at q < 0.05). Download Figure 4-5, TIF file. [file jneuro-44-e1856232024-s008.tif]

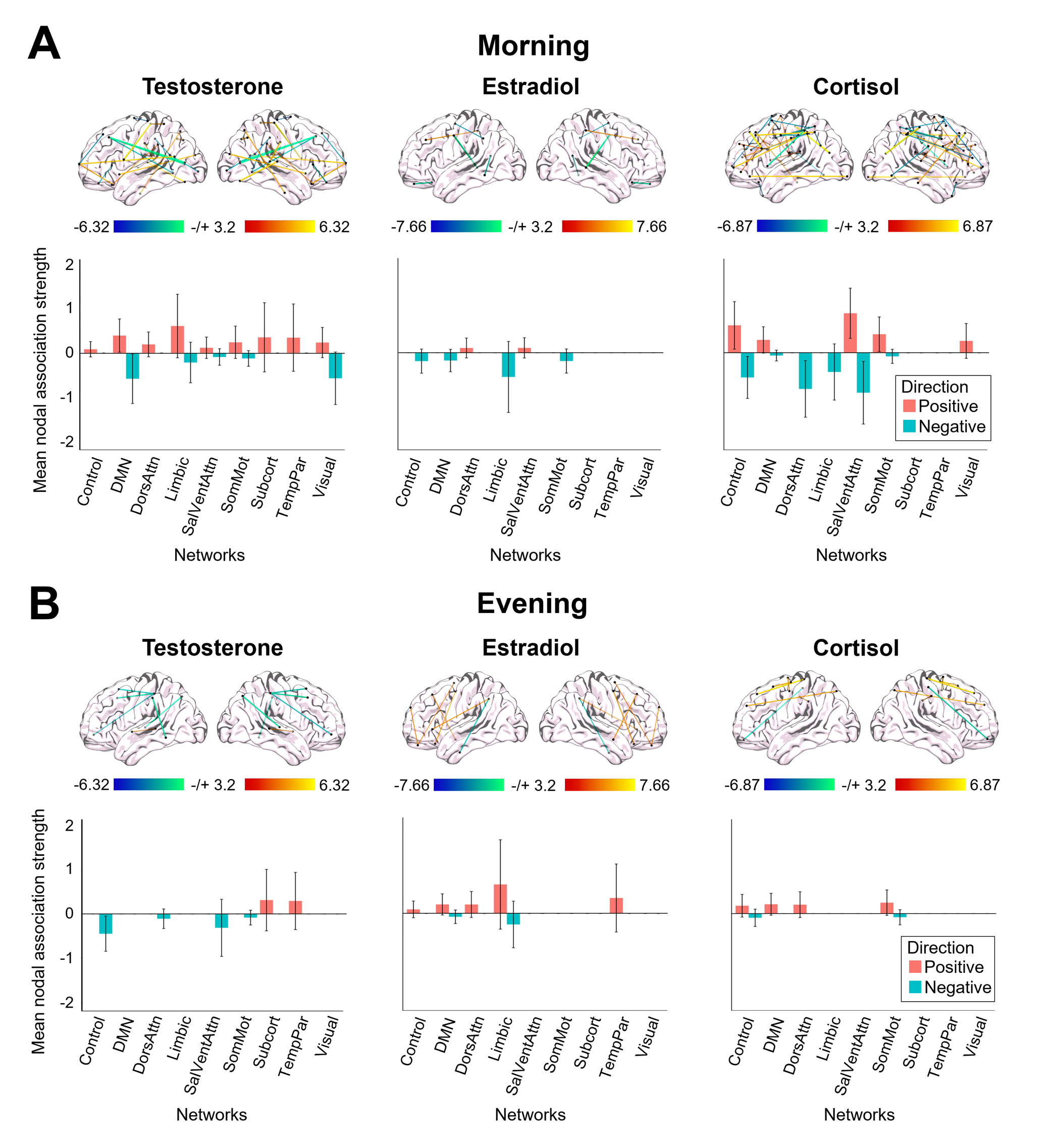

Supplement: Figure 4-6 — Morning and evening sessions show different patterns of whole-brain connectivity. Steroid hormone concentrations show different patterns of whole-brain coherence and mean nodal association strengths in the morning sessions (A) compared to evening sessions (B). Time-synchronous associations between total testosterone, estradiol, and cortisol and coherence (top), and mean nodal association strengths by network (bottom). All edges and mean nodal association strengths are corrected for multiple comparisons (FDR at q < 0.05). Download Figure 4-6, TIF file. [file jneuro-44-e1856232024-s009.tif]
